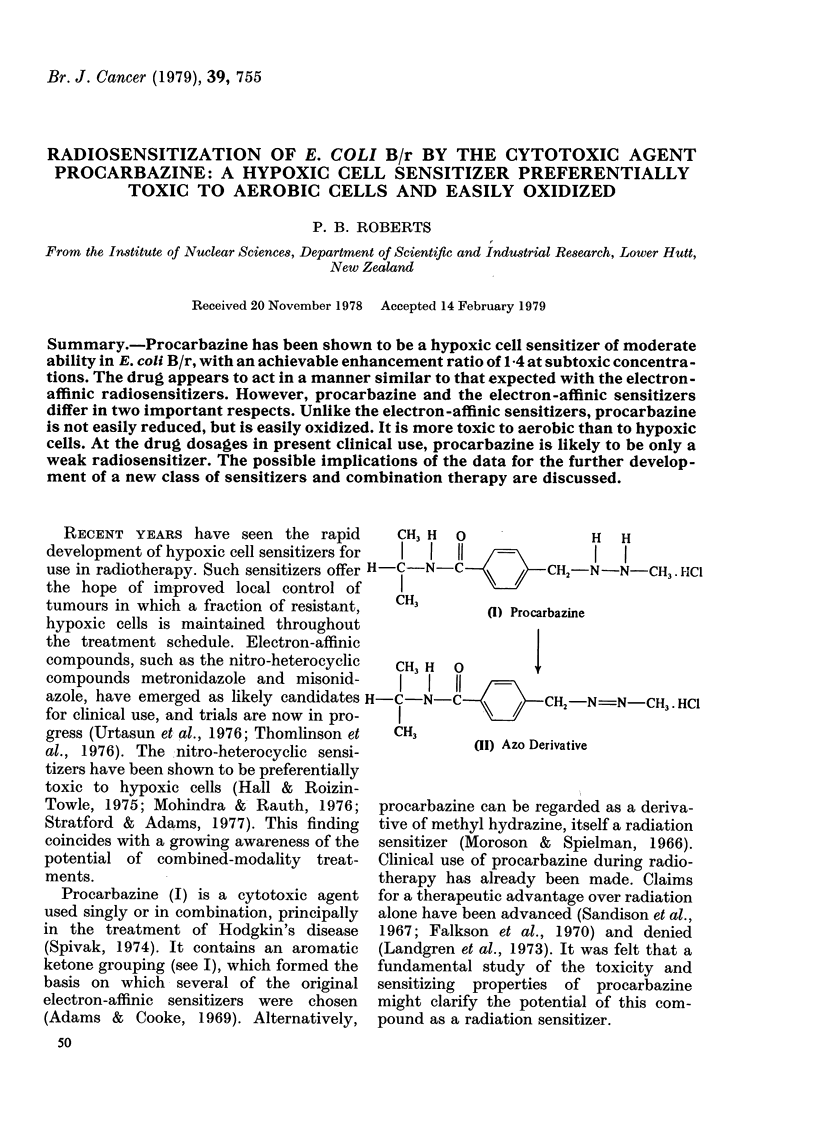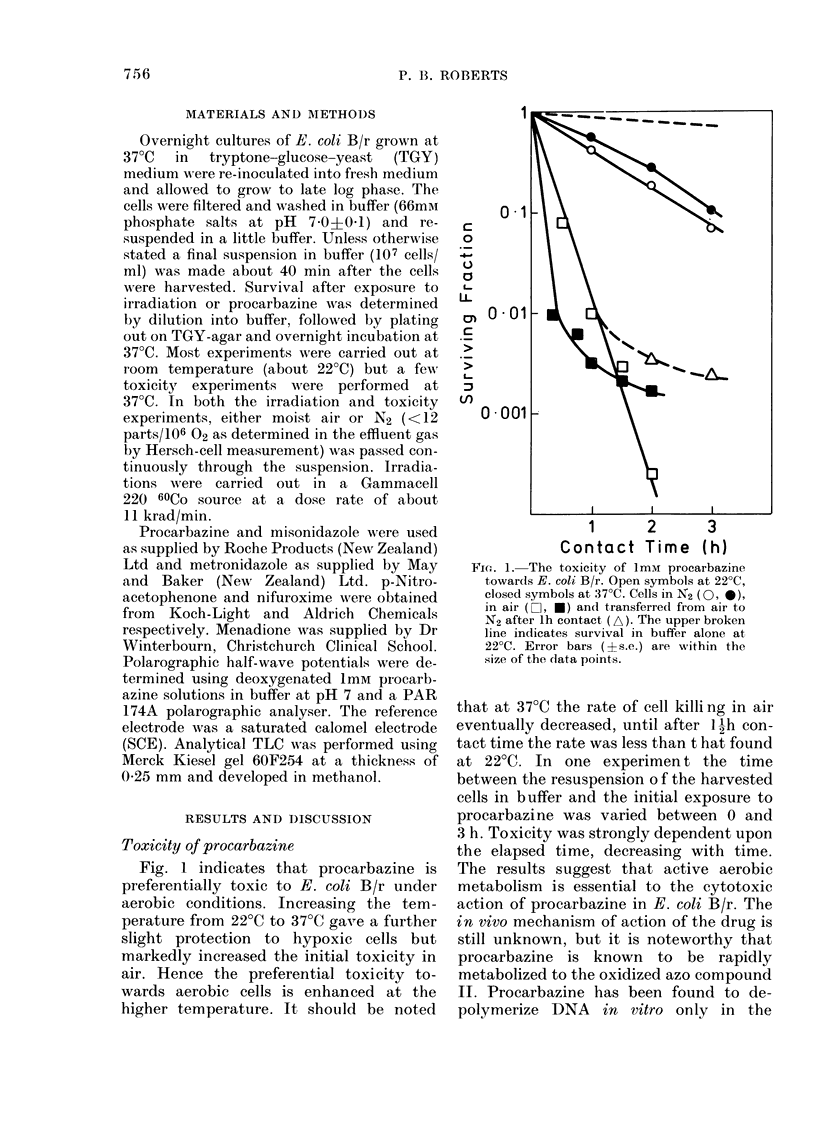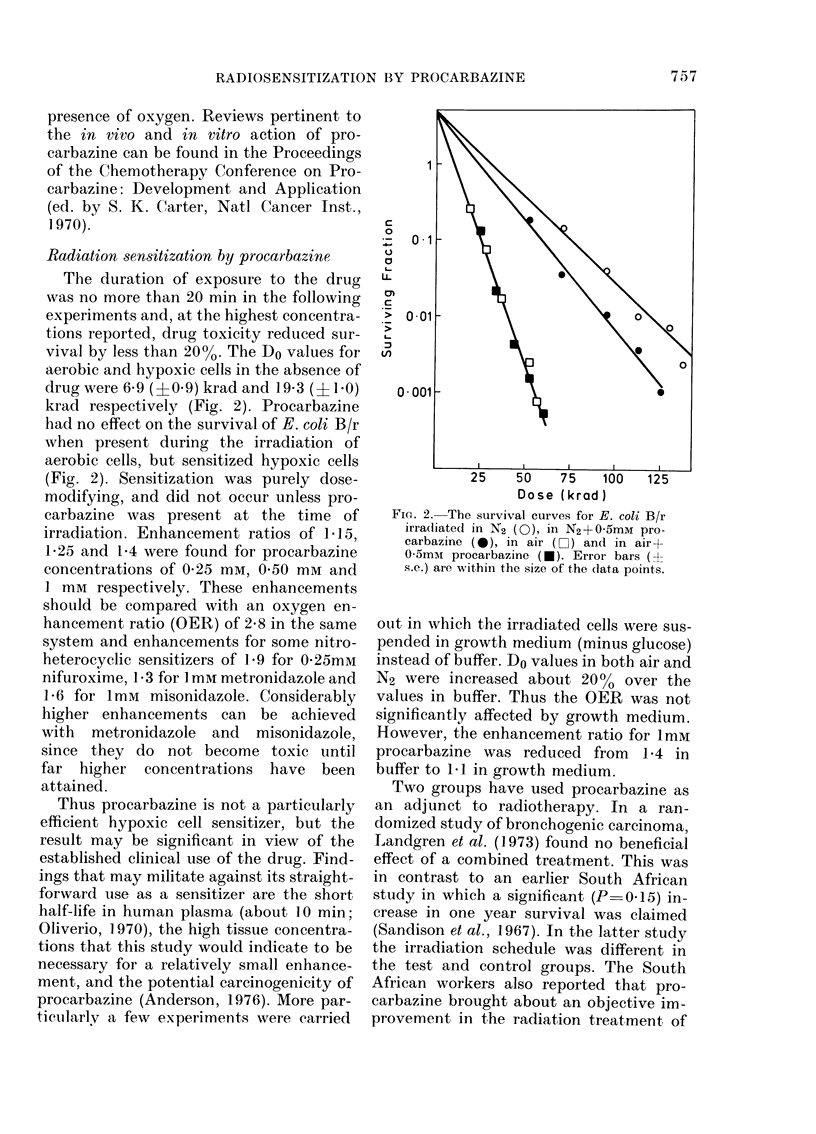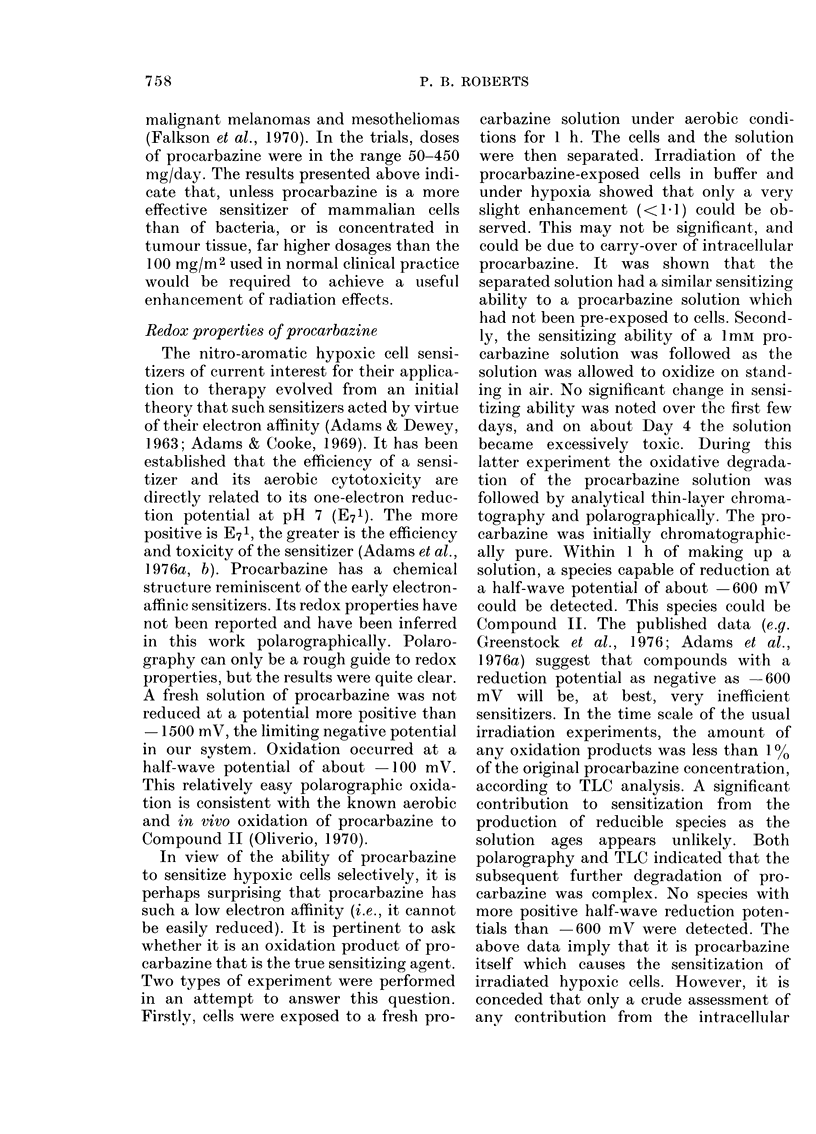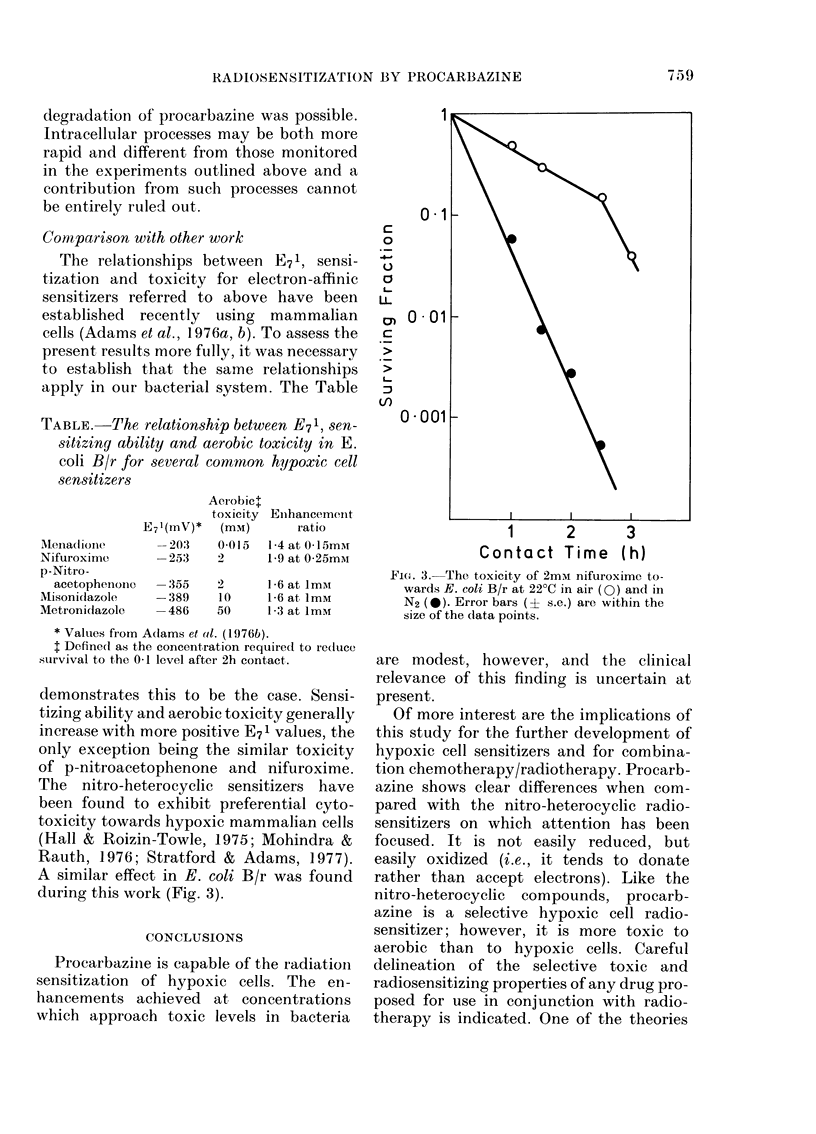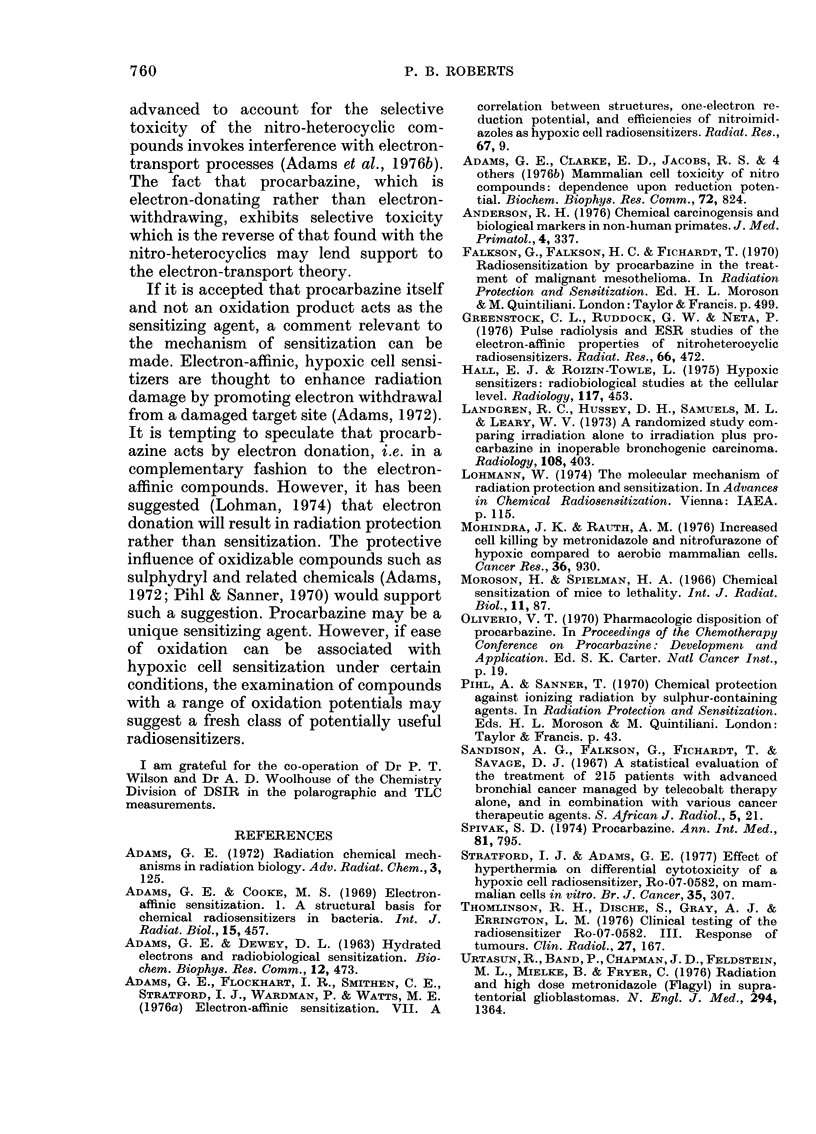# Radiosensitization of E. coli B/r by the cytotoxic agent procarbazine: a hypoxic cell sensitizer preferentially toxic to aerobic cells and easily oxidized.

**DOI:** 10.1038/bjc.1979.129

**Published:** 1979-06

**Authors:** P. B. Roberts

## Abstract

Procarbazine has been shown to be a hypoxic cell sensitizer of moderate ability in E. coli B/r, with an achievable enhancement ratio of 1.4 at subtoxic concentrations. The drug appears to act in a manner similar to the expected with the electron-affinic radiosensitizers. However, procarbazine and the electron-affinic sensitizers differ in two important respects. Unlike the electron-affinic sensitizers, procarbazine is not easily reduced, but is easily oxidized. It is more toxic to aerobic than to hypoxic cells. At the drug dosages in present clinical use, procarbazine is likely to be only a weak radiosensitizer. The possible implications of the data for the further development of a new class of sensitizers and combination therapy are discussed.


*Br. J. Cancer* (1979), **39**, 755

### RADIOSENSITIZATION OF *E. COLI* B/r BY THE CYTOTOXIC AGENT PROCARBAZINE: A HYPOXIC CELL SENSITIZER PREFERENTIALLY TOXIC TO AEROBIC CELLS AND EASILY OXIDIZED

P. B. ROBERTS

*From the Institute of Nuclear Sciences, Department of Scientific and Industrial Research, Lower Hutt, New Zealand*

Received 20 November 1978 Accepted 14 February 1979

**Summary.—Procarbazine has been shown to be a hypoxic cell sensitizer of moderate ability in *E. coli* B/r, with an achievable enhancement ratio of 1·4 at subtoxic concentrations. The drug appears to act in a manner similar to that expected with the electron-affinic radiosensitizers. However, procarbazine and the electron-affinic sensitizers differ in two important respects. Unlike the electron-affinic sensitizers, procarbazine is not easily reduced, but is easily oxidized. It is more toxic to aerobic than to hypoxic cells. At the drug dosages in present clinical use, procarbazine is likely to be only a weak radiosensitizer. The possible implications of the data for the further development of a new class of sensitizers and combination therapy are discussed.**

Recent years have seen the rapid development of hypoxic cell sensitizers for use in radiotherapy. Such sensitizers offer the hope of improved local control of tumours in which a fraction of resistant, hypoxic cells is maintained throughout the treatment schedule. Electron-affinic compounds, such as the nitro-heterocyclic compounds metronidazole and misonidazole, have emerged as likely candidates for clinical use, and trials are now in progress (Urtasun *et al.*, 1976; Thomlinson *et al.*, 1976). The nitro-heterocyclic sensitizers have been shown to be preferentially toxic to hypoxic cells (Hall & Roizin-Towle, 1975; Mohindra & Rauth, 1976; Stratford & Adams, 1977). This finding coincides with a growing awareness of the potential of combined-modality treatments.

(I) Procarbazine

(II) Azo Derivative

Procarbazine (I) is a cytotoxic agent used singly or in combination, principally in the treatment of Hodgkin's disease (Spivak, 1974). It contains an aromatic ketone grouping (see I), which formed the basis on which several of the original electron-affinic sensitizers were chosen (Adams & Cooke, 1969). Alternatively, procarbazine can be regarded as a derivative of methyl hydrazine, itself a radiation sensitizer (Moroson & Spielman, 1966). Clinical use of procarbazine during radiotherapy has already been made. Claims for a therapeutic advantage over radiation alone have been advanced (Sandison *et al.*, 1967; Falkson *et al.*, 1970) and denied (Landgren *et al.*, 1973). It was felt that a fundamental study of the toxicity and sensitizing properties of procarbazine might clarify the potential of this compound as a radiation sensitizer.

### MATERIALS AND METHODS

Overnight cultures of *E. coli* B/r grown at 37°C in tryptone–glucose–yeast (TGY) medium were re-inoculated into fresh medium and allowed to grow to late log phase. The cells were filtered and washed in buffer (66mM phosphate salts at pH 7·0±0·1) and re-suspended in a little buffer. Unless otherwise stated a final suspension in buffer ($10^7$ cells/ml) was made about 40 min after the cells were harvested. Survival after exposure to irradiation or procarbazine was determined by dilution into buffer, followed by plating out on TGY-agar and overnight incubation at 37°C. Most experiments were carried out at room temperature (about 22°C) but a few toxicity experiments were performed at 37°C. In both the irradiation and toxicity experiments, either moist air or $N_2$ (<12 parts/$10^6$ $O_2$ as determined in the effluent gas by Hersch-cell measurement) was passed continuously through the suspension. Irradiations were carried out in a Gammacell 220 $^{60}Co$ source at a dose rate of about 11 krad/min.

Procarbazine and misonidazole were used as supplied by Roche Products (New Zealand) Ltd and metronidazole as supplied by May and Baker (New Zealand) Ltd. p-Nitro-acetophenone and nifuroxime were obtained from Koch-Light and Aldrich Chemicals respectively. Menadione was supplied by Dr Winterbourn, Christchurch Clinical School. Polarographic half-wave potentials were determined using deoxygenated 1mM procarbazine solutions in buffer at pH 7 and a PAR 174A polarographic analyser. The reference electrode was a saturated calomel electrode (SCE). Analytical TLC was performed using Merck Kiesel gel 60F254 at a thickness of 0·25 mm and developed in methanol.

FIG. 1.—The toxicity of 1mM procarbazine towards *E. coli* B/r. Open symbols at 22°C, closed symbols at 37°C. Cells in $N_2$ (○, ●), in air (□, ■) and transferred from air to $N_2$ after 1h contact (△). The upper broken line indicates survival in buffer alone at 22°C. Error bars (±s.e.) are within the size of the data points.

### RESULTS AND DISCUSSION

### *Toxicity of procarbazine*

Fig. 1 indicates that procarbazine is preferentially toxic to *E. coli* B/r under aerobic conditions. Increasing the temperature from 22°C to 37°C gave a further slight protection to hypoxic cells but markedly increased the initial toxicity in air. Hence the preferential toxicity towards aerobic cells is enhanced at the higher temperature. It should be noted that at 37°C the rate of cell killing in air eventually decreased, until after 1½h contact time the rate was less than that found at 22°C. In one experiment the time between the resuspension of the harvested cells in buffer and the initial exposure to procarbazine was varied between 0 and 3 h. Toxicity was strongly dependent upon the elapsed time, decreasing with time. The results suggest that active aerobic metabolism is essential to the cytotoxic action of procarbazine in *E. coli* B/r. The *in vivo* mechanism of action of the drug is still unknown, but it is noteworthy that procarbazine is known to be rapidly metabolized to the oxidized azo compound II. Procarbazine has been found to depolymerize DNA *in vitro* only in the presence of oxygen. Reviews pertinent to the *in vivo* and *in vitro* action of procarbazine can be found in the Proceedings of the Chemotherapy Conference on Procarbazine: Development and Application (ed. by S. K. Carter, Natl Cancer Inst., 1970).

*Radiation sensitization by procarbazine*

The duration of exposure to the drug was no more than 20 min in the following experiments and, at the highest concentrations reported, drug toxicity reduced survival by less than 20%. The $D_0$ values for aerobic and hypoxic cells in the absence of drug were 6·9 (±0·9) krad and 19·3 (±1·0) krad respectively (Fig. 2). Procarbazine had no effect on the survival of *E. coli* B/r when present during the irradiation of aerobic cells, but sensitized hypoxic cells (Fig. 2). Sensitization was purely dose-modifying, and did not occur unless procarbazine was present at the time of irradiation. Enhancement ratios of 1·15, 1·25 and 1·4 were found for procarbazine concentrations of 0·25 mM, 0·50 mM and 1 mM respectively. These enhancements should be compared with an oxygen enhancement ratio (OER) of 2·8 in the same system and enhancements for some nitroheterocyclic sensitizers of 1·9 for 0·25mM nifuroxime, 1·3 for 1mM metronidazole and 1·6 for 1mM misonidazole. Considerably higher enhancements can be achieved with metronidazole and misonidazole, since they do not become toxic until far higher concentrations have been attained.

Thus procarbazine is not a particularly efficient hypoxic cell sensitizer, but the result may be significant in view of the established clinical use of the drug. Findings that may militate against its straightforward use as a sensitizer are the short half-life in human plasma (about 10 min; Oliverio, 1970), the high tissue concentrations that this study would indicate to be necessary for a relatively small enhancement, and the potential carcinogenicity of procarbazine (Anderson, 1976). More particularly a few experiments were carried out in which the irradiated cells were suspended in growth medium (minus glucose) instead of buffer. $D_0$ values in both air and $N_2$ were increased about 20% over the values in buffer. Thus the OER was not significantly affected by growth medium. However, the enhancement ratio for 1mM procarbazine was reduced from 1·4 in buffer to 1·1 in growth medium.

FIG. 2.—The survival curves for *E. coli* B/r irradiated in $N_2$ (○), in $N_2$+0·5mM procarbazine (●), in air (□) and in air+0·5mM procarbazine (■). Error bars (± s.e.) are within the size of the data points.

Two groups have used procarbazine as an adjunct to radiotherapy. In a randomized study of bronchogenic carcinoma, Landgren *et al.* (1973) found no beneficial effect of a combined treatment. This was in contrast to an earlier South African study in which a significant ($P$=0·15) increase in one year survival was claimed (Sandison *et al.*, 1967). In the latter study the irradiation schedule was different in the test and control groups. The South African workers also reported that procarbazine brought about an objective improvement in the radiation treatment of malignant melanomas and mesotheliomas (Falkson *et al.*, 1970). In the trials, doses of procarbazine were in the range 50–450 mg/day. The results presented above indicate that, unless procarbazine is a more effective sensitizer of mammalian cells than of bacteria, or is concentrated in tumour tissue, far higher dosages than the 100 mg/m$^2$ used in normal clinical practice would be required to achieve a useful enhancement of radiation effects.

*Redox properties of procarbazine*

The nitro-aromatic hypoxic cell sensitizers of current interest for their application to therapy evolved from an initial theory that such sensitizers acted by virtue of their electron affinity (Adams & Dewey, 1963; Adams & Cooke, 1969). It has been established that the efficiency of a sensitizer and its aerobic cytotoxicity are directly related to its one-electron reduction potential at pH 7 ($E_7^1$). The more positive is $E_7^1$, the greater is the efficiency and toxicity of the sensitizer (Adams *et al.*, 1976*a*, *b*). Procarbazine has a chemical structure reminiscent of the early electron-affinic sensitizers. Its redox properties have not been reported and have been inferred in this work polarographically. Polarography can only be a rough guide to redox properties, but the results were quite clear. A fresh solution of procarbazine was not reduced at a potential more positive than $-1500$ mV, the limiting negative potential in our system. Oxidation occurred at a half-wave potential of about $-100$ mV. This relatively easy polarographic oxidation is consistent with the known aerobic and *in vivo* oxidation of procarbazine to Compound II (Oliverio, 1970).

In view of the ability of procarbazine to sensitize hypoxic cells selectively, it is perhaps surprising that procarbazine has such a low electron affinity (*i.e.*, it cannot be easily reduced). It is pertinent to ask whether it is an oxidation product of procarbazine that is the true sensitizing agent. Two types of experiment were performed in an attempt to answer this question. Firstly, cells were exposed to a fresh procarbazine solution under aerobic conditions for 1 h. The cells and the solution were then separated. Irradiation of the procarbazine-exposed cells in buffer and under hypoxia showed that only a very slight enhancement ($<1{\cdot}1$) could be observed. This may not be significant, and could be due to carry-over of intracellular procarbazine. It was shown that the separated solution had a similar sensitizing ability to a procarbazine solution which had not been pre-exposed to cells. Secondly, the sensitizing ability of a 1mM procarbazine solution was followed as the solution was allowed to oxidize on standing in air. No significant change in sensitizing ability was noted over the first few days, and on about Day 4 the solution became excessively toxic. During this latter experiment the oxidative degradation of the procarbazine solution was followed by analytical thin-layer chromatography and polarographically. The procarbazine was initially chromatographically pure. Within 1 h of making up a solution, a species capable of reduction at a half-wave potential of about $-600$ mV could be detected. This species could be Compound II. The published data (*e.g.* Greenstock *et al.*, 1976; Adams *et al.*, 1976*a*) suggest that compounds with a reduction potential as negative as $-600$ mV will be, at best, very inefficient sensitizers. In the time scale of the usual irradiation experiments, the amount of any oxidation products was less than 1% of the original procarbazine concentration, according to TLC analysis. A significant contribution to sensitization from the production of reducible species as the solution ages appears unlikely. Both polarography and TLC indicated that the subsequent further degradation of procarbazine was complex. No species with more positive half-wave reduction potentials than $-600$ mV were detected. The above data imply that it is procarbazine itself which causes the sensitization of irradiated hypoxic cells. However, it is conceded that only a crude assessment of any contribution from the intracellular degradation of procarbazine was possible. Intracellular processes may be both more rapid and different from those monitored in the experiments outlined above and a contribution from such processes cannot be entirely ruled out.

*Comparison with other work*

The relationships between $E_7^1$, sensitization and toxicity for electron-affinic sensitizers referred to above have been established recently using mammalian cells (Adams *et al.*, 1976*a*, *b*). To assess the present results more fully, it was necessary to establish that the same relationships apply in our bacterial system. The Table

TABLE.—*The relationship between $E_7^1$, sensitizing ability and aerobic toxicity in* E. coli *B/r for several common hypoxic cell sensitizers*

| | $E_7^1$(mV)* | Aerobic‡ toxicity (mM) | Enhancement ratio |
|---|---|---|---|
| Menadione | −203 | 0·015 | 1·4 at 0·15mM |
| Nifuroxime | −253 | 2 | 1·9 at 0·25mM |
| p-Nitro-acetophenone | −355 | 2 | 1·6 at 1mM |
| Misonidazole | −389 | 10 | 1·6 at 1mM |
| Metronidazole | −486 | 50 | 1·3 at 1mM |

* Values from Adams *et al.* (1976*b*).
‡ Defined as the concentration required to reduce survival to the 0·1 level after 2h contact.

demonstrates this to be the case. Sensitizing ability and aerobic toxicity generally increase with more positive $E_7^1$ values, the only exception being the similar toxicity of p-nitroacetophenone and nifuroxime. The nitro-heterocyclic sensitizers have been found to exhibit preferential cytotoxicity towards hypoxic mammalian cells (Hall & Roizin-Towle, 1975; Mohindra & Rauth, 1976; Stratford & Adams, 1977). A similar effect in *E. coli* B/r was found during this work (Fig. 3).

FIG. 3.—The toxicity of 2mM nifuroxime towards *E. coli* B/r at 22°C in air (○) and in $N_2$ (●). Error bars (± s.e.) are within the size of the data points.

### CONCLUSIONS

Procarbazine is capable of the radiation sensitization of hypoxic cells. The enhancements achieved at concentrations which approach toxic levels in bacteria are modest, however, and the clinical relevance of this finding is uncertain at present.

Of more interest are the implications of this study for the further development of hypoxic cell sensitizers and for combination chemotherapy/radiotherapy. Procarbazine shows clear differences when compared with the nitro-heterocyclic radiosensitizers on which attention has been focused. It is not easily reduced, but easily oxidized (*i.e.*, it tends to donate rather than accept electrons). Like the nitro-heterocyclic compounds, procarbazine is a selective hypoxic cell radiosensitizer; however, it is more toxic to aerobic than to hypoxic cells. Careful delineation of the selective toxic and radiosensitizing properties of any drug proposed for use in conjunction with radiotherapy is indicated. One of the theories advanced to account for the selective toxicity of the nitro-heterocyclic compounds invokes interference with electron-transport processes (Adams *et al.*, 1976*b*). The fact that procarbazine, which is electron-donating rather than electron-withdrawing, exhibits selective toxicity which is the reverse of that found with the nitro-heterocyclics may lend support to the electron-transport theory.

If it is accepted that procarbazine itself and not an oxidation product acts as the sensitizing agent, a comment relevant to the mechanism of sensitization can be made. Electron-affinic, hypoxic cell sensitizers are thought to enhance radiation damage by promoting electron withdrawal from a damaged target site (Adams, 1972). It is tempting to speculate that procarbazine acts by electron donation, *i.e.* in a complementary fashion to the electron-affinic compounds. However, it has been suggested (Lohman, 1974) that electron donation will result in radiation protection rather than sensitization. The protective influence of oxidizable compounds such as sulphydryl and related chemicals (Adams, 1972; Pihl & Sanner, 1970) would support such a suggestion. Procarbazine may be a unique sensitizing agent. However, if ease of oxidation can be associated with hypoxic cell sensitization under certain conditions, the examination of compounds with a range of oxidation potentials may suggest a fresh class of potentially useful radiosensitizers.

I am grateful for the co-operation of Dr P. T. Wilson and Dr A. D. Woolhouse of the Chemistry Division of DSIR in the polarographic and TLC measurements.

### REFERENCES

ADAMS, G. E. (1972) Radiation chemical mechanisms in radiation biology. *Adv. Radiat. Chem.*, **3**, 125.

ADAMS, G. E. & COOKE, M. S. (1969) Electron-affinic sensitization. 1. A structural basis for chemical radiosensitizers in bacteria. *Int. J. Radiat. Biol.*, **15**, 457.

ADAMS, G. E. & DEWEY, D. L. (1963) Hydrated electrons and radiobiological sensitization. *Biochem. Biophys. Res. Comm.*, **12**, 473.

ADAMS, G. E., FLOCKHART, I. R., SMITHEN, C. E., STRATFORD, I. J., WARDMAN, P. & WATTS, M. E. (1976*a*) Electron-affinic sensitization. VII. A correlation between structures, one-electron reduction potential, and efficiencies of nitroimidazoles as hypoxic cell radiosensitizers. *Radiat. Res.*, **67**, 9.

ADAMS, G. E., CLARKE, E. D., JACOBS, R. S. & 4 others (1976*b*) Mammalian cell toxicity of nitro compounds: dependence upon reduction potential. *Biochem. Biophys. Res. Comm.*, **72**, 824.

ANDERSON, R. H. (1976) Chemical carcinogensis and biological markers in non-human primates. *J. Med. Primatol.*, **4**, 337.

FALKSON, G., FALKSON, H. C. & FICHARDT, T. (1970) Radiosensitization by procarbazine in the treatment of malignant mesothelioma. In *Radiation Protection and Sensitization*. Ed. H. L. Moroson & M. Quintiliani. London: Taylor & Francis. p. 499.

GREENSTOCK, C. L., RUDDOCK, G. W. & NETA, P. (1976) Pulse radiolysis and ESR studies of the electron-affinic properties of nitroheterocyclic radiosensitizers. *Radiat. Res.*, **66**, 472.

HALL, E. J. & ROIZIN-TOWLE, L. (1975) Hypoxic sensitizers: radiobiological studies at the cellular level. *Radiology*, **117**, 453.

LANDGREN, R. C., HUSSEY, D. H., SAMUELS, M. L. & LEARY, W. V. (1973) A randomized study comparing irradiation alone to irradiation plus procarbazine in inoperable bronchogenic carcinoma. *Radiology*, **108**, 403.

LOHMANN, W. (1974) The molecular mechanism of radiation protection and sensitization. In *Advances in Chemical Radiosensitization*. Vienna: IAEA. p. 115.

MOHINDRA, J. K. & RAUTH, A. M. (1976) Increased cell killing by metronidazole and nitrofurazone of hypoxic compared to aerobic mammalian cells. *Cancer Res.*, **36**, 930.

MOROSON, H. & SPIELMAN, H. A. (1966) Chemical sensitization of mice to lethality. *Int. J. Radiat. Biol.*, **11**, 87.

OLIVERIO, V. T. (1970) Pharmacologic disposition of procarbazine. In *Proceedings of the Chemotherapy Conference on Procarbazine: Developmenı and Application*. Ed. S. K. Carter. *Natl Cancer Inst.*, p. 19.

PIHL, A. & SANNER, T. (1970) Chemical protection against ionizing radiation by sulphur-containing agents. In *Radiation Protection and Sensitization*. Eds. H. L. Moroson & M. Quintiliani. London: Taylor & Francis. p. 43.

SANDISON, A. G., FALKSON, G., FICHARDT, T. & SAVAGE, D. J. (1967) A statistical evaluation of the treatment of 215 patients with advanced bronchial cancer managed by telecobalt therapy alone, and in combination with various cancer therapeutic agents. *S. African J. Radiol.*, **5**, 21.

SPIVAK, S. D. (1974) Procarbazine. *Ann. Int. Med.*, **81**, 795.

STRATFORD, I. J. & ADAMS, G. E. (1977) Effect of hyperthermia on differential cytotoxicity of a hypoxic cell radiosensitizer, Ro-07-0582, on mammalian cells *in vitro*. *Br. J. Cancer*, **35**, 307.

THOMLINSON, R. H., DISCHE, S., GRAY, A. J. & ERRINGTON, L. M. (1976) Clinical testing of the radiosensitizer Ro-07-0582. III. Response of tumours. *Clin. Radiol.*, **27**, 167.

URTASUN, R., BAND, P., CHAPMAN, J. D., FELDSTEIN, M. L., MIELKE, B. & FRYER, C. (1976) Radiation and high dose metronidazole (Flagyl) in supratentorial glioblastomas. *N. Engl. J. Med.*, **294**, 1364.